# Normalized lifespan inequality: disentangling the longevity–lifespan variability nexus

**DOI:** 10.1186/s41118-021-00150-6

**Published:** 2022-01-10

**Authors:** Iñaki Permanyer, Jiaxin Shi

**Affiliations:** 1 Centre d’Estudis Demogràfics, Barcelona, Spain; 2 ICREA, Passeig Lluís Companys 23, 08010 Barcelona, Spain; 3 Max Planck Institute for Demographic Research, Rostock, Germany; 4 Leverhulme Centre for Demographic Science, Department of Sociology, University of Oxford, Oxford, UK

**Keywords:** Lifespan inequality, Life expectancy, Maximal lifespan record, Mortality

## Abstract

Previous studies have documented a historically strong and negative association between countries’ life expectancy (i.e., average longevity) and length-of-life inequality (i.e., variability in ages at death). The relationship between both variables might be partially explained by life expectancy increasing at a faster pace than maximal length of life, a phenomenon that mechanically compresses the age-at-death distribution and has not been taken into consideration in previous studies. In this paper, we propose a new approach to lifespan inequality measurement that accounts for the (uncertainly) bounded nature of length-of-life. Applying the new approach to the countries of the Human Mortality Database, we observe that the decline in overall lifespan variability typically associated with increases in longevity seems to stop and even reverse at higher levels of life expectancy. This suggests the emergence of worrying ethical dilemmas, whereby higher achievements in longevity would only be possible at the expense of higher lifespan variability.

## Introduction

The past decades have witnessed an increasing interest in understanding the variation in length of life among demographers and researchers from other disciplines (see, among others, Aburto & Van Raalte, 2018; Colchero et al., 2016; Edwards, 2011; Edwards & Tuljapurkar, 2005; Engelman et al., 2010; Gillespie et al., 2014; Nau & Firebaugh, 2012; Seaman et al., 2019; Seligman et al., 2016; Smits & Monden, 2009; Van Raalte & Caswell, 2013; Van Raalte et al., 2014, 2018; Vaupel et al., 2011; Wilmoth & Horiuchi, 1999). Variation in length of life is one of the most fundamental inequalities in human populations; reducing such inequalities and enhancing long lives across all sectors of the population have become a prominent issue on global research and policy agendas. Inequalities in length of life are not just a consequence of natural stochasticity, but may indicate the systematically unequal distribution of resources embedded in our society. Additionally, higher levels of lifespan inequality imply higher uncertainty in the timing of death—an issue with enduring impact on individuals’ well-being and influencing important decisions during their life courses (e.g., investing in higher education, childbearing decisions, applying for a mortgage, contracting or upgrading a health insurance policy, or saving for retirement; see Edwards, 2013).

Studies have identified a historically strong and negative association between life expectancy (i.e., average longevity) and length-of-life inequality^1^ (Colchero et al., 2016; Edwards, 2011; Permanyer & Scholl, 2019; Smits & Monden, 2009; Vaupel et al., 2011, 2021; Wilmoth & Horiuchi, 1999). The mechanisms explaining such association have received considerable attention from recent demographic research. For instance, Aburto and colleagues (2020) showed that the association was stronger when mortality reduction occurred more at younger ages, and weaker when mortality improvement was more observed at older ages. Nigri and colleagues (2021a, 2021b) found that the strength of this association is related to the location and speed when a country moves along the life expectancy distribution.

**Fig. 1:**
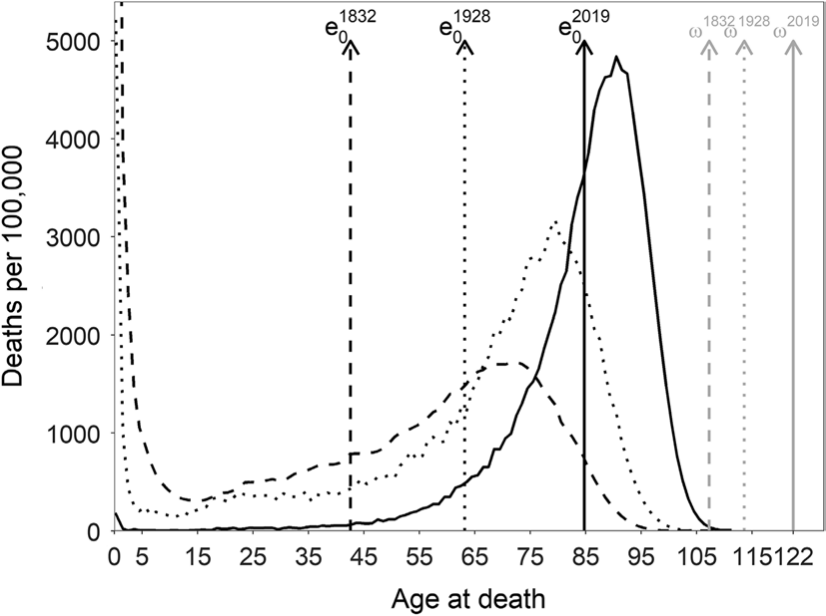
Age-at-death distributions for Swedish females in 1832 (dashed), 1928 (dotted), and 2019 (solid). Black and grey vertical lines refer to life expectancy and record lifespan up until that year, respectively (Source: Authors’ elaboration based on the HMD data)

We offer a different perspective. To illustrate why such relationship is so strong and negative, Fig. 1 shows the length-of-life distributions for Swedish females in 3 years, together with the life expectancies (${e}_{0}$) and the worldwide human lifespan record ($\omega$) for the corresponding years. The first time point is 1832 (the first year with available data), and the last one is 2019 (the most recent available year for Sweden from the Human Mortality Database (HMD)). Halfway between them, we also show the results for 1928, a year when a new record maximal age was registered. The first distribution is characteristic of a high mortality setting, with high infant and child mortality and low life expectancy levels, and the last one is characteristic of a high-income country that has passed the initial stages of the epidemiological transition (Omran, 1971; Vallin & Meslé, 2004) and has extremely low levels of child and early adult mortality. As can be inferred from the graph, the average of the distribution (i.e., life expectancy) increases faster than the maximal lifespan. The ratio between these quantities was 0.40 in 1832, 0.56 in 1928, and 0.69 in 2019. Over time, life expectancy gradually approaches the human lifespan record (both in absolute and relative terms); as a result, the distribution is increasingly squeezed within a shorter age range, thus reducing its variability. These general trends are not exclusive to Swedish women. They have been observed in the majority of countries with available data, for both women and men.

In this setting, it is not surprising that lifespan inequality tends to decrease as life expectancy approaches the maximal lifespan. Given the mechanically driven appearance of such relationship, in this paper we ask: could it have been otherwise? Put differently, could lifespan inequality have behaved differently when life expectancy gradually approaches the maximal lifespan, or is the strong relationship between both variables an artifact of the way in which inequality is measured? This is a question with important theoretical and practical implications. From a theoretical perspective, studying the relationship between average longevity and lifespan variability is fundamental for a proper understanding of the dynamics in human mortality, and is crucial for forecasting future patterns of population health (see related work by Bohk-Ewald et al., 2017; Nigri et al., 2021a, 2021b; Rabbi & Mazzuco, 2021). From a practical and policy-making perspective, it is very important to know whether and to what extent the normatively desirable goals of increasing longevity *and* reducing lifespan variability can be achieved simultaneously or if one can only be attained to the detriment of the other (Benach et al., 2011, 2013; Smits & Monden, 2009; Vaupel et al., 2011). The fact that longevity and lifespan variability are so strongly connected raises several issues. First, it might be argued that it is not immediately clear that studying lifespan inequality can provide new insights beyond what we already know from studying life expectancy alone. Second, the comparison of lifespan inequalities for distributions with very different life expectancy levels is compromised. Alongside these issues, there is the unresolved debate of whether inequality should be measured in absolute or relative terms, a decision that depends on normative values and which might lead to inconsistent and conflicting conclusions (Asada, 2010; Clarke et al., 2002; Erreygers & van Ourti, 2011; Houweling et al., 2007; Mackenbach, 2015).

Therefore, we propose a new approach to lifespan inequality measurement that, unlike existing measures, explicitly takes into consideration the shrinking room for variability that ensues when both life expectancy and maximal lifespan get closer over time. The idea is quite simple: we propose a new normalization procedure that compares observed inequality with respect to the maximal inequality one could possibly observe in a hypothetical distribution having the same mean. In this way, we obtain a normalized index of inequality, measuring how far we are from the inequality-maximizing scenario. As shown below, this approach assumes that individuals’ length of life cannot exceed the maximal lifespan $\omega$. Given the uncertainty surrounding that value, we develop simple methods to investigate the robustness of any statement one might want to make with regard to alternative values of $\omega$.

The role of a maximal lifespan (or to quote Myers and Manton (1984): the “biological limits on normal life span of human species”) in determining changes in length-of-life variation has long been acknowledged by researchers. Although the upper boundary is sometimes with respect to life expectancy at the population level (Fries, 1980), individual-level maximal lifespan is implied. Because of such a limit, both morbidity and mortality tend to be pushed into a smaller age range (Cheung et al., 2005; Fries, 1980; Wilmoth & Horiuchi, 1999). The observed decreasing trends of lifespan variability together with increasing life expectancy in the past decades generally agree with this theoretical prediction. Unlike prior research (which *implicitly* assumed that the observed relationship between both variables could be driven by the existence of such upper bound), we *explicitly* incorporate the maximal lifespan $\omega$ as a key ingredient that could be taken into consideration when measuring lifespan inequality.

With our approach, we may see patterns that may have been hidden in common inequality measures. First, the steady decline in lifespan inequality as measured by conventional measures may no longer hold, particularly for countries with high levels of longevity. Second, as our approach explicitly takes the level of life expectancy into account (see below), life expectancy may be less correlated with the normalized inequality than with common inequality measures. Third, the normalization process can impact differently on different countries, which might lead to alternative country rankings of lifespan inequality, as compared to conventional approaches. Lastly, although our focus is not on the limit of lifespan (e.g., whether it exists, or what it exactly is), the new measure relies on such a limit. Therefore, we expect the choice of the limit may lead to different results, for instance, different country rankings.

## Measurement and definitions

In this section, we introduce the notations. Life table age-at-death distributions are generically denoted with capital letters, such as $A, B$ or $C$.^2^ Ages range from 0 to $\omega$. For each age-at-death distribution, the vector ${\varvec{d}}=\{{d}_{0},{d}_{1},\dots ,{d}_{\omega -1},{d}_{\omega }\}$ counts the relative share of deaths occurring at different ages. Since ${\sum }_{x}{d}_{x}=1$, each vector ${\varvec{d}}$ represents a density function over the age-at-death distribution. Let ${D}^{\omega }$ denote the set of all age-at-death distributions. For each age-at-death distribution $A\in {D}^{\omega }$, ${e}_{0}\left(A\right)$ denotes the corresponding mean age at death (i.e., the life expectancy at birth).

An inequality index $I$ is *absolute* if it remains unaffected when the same quantity is added to all elements of the distribution (i.e., the length of life of all individuals is lengthened by the same amount and the age-at-death distribution is translated). Analogously, an inequality index $I$ is *relative* if it remains unaffected when all elements of the distribution are scaled by the same proportionality factor. The choice between absolute and relative indices is a matter of subjective assessment that depends on the evaluator’s value judgments about inequality equivalence (Atkinson, 2013)—an issue that has led to a long and inconclusive debate. Currently, absolute and relative inequality measures have been used indistinctly in the literature of length-of-life inequality, with numerous examples on both sides.^3^

We now list the inequality indices that will be used in this paper and which have also been widely applied in the literature (Permanyer & Scholl, 2019; Shkolnikov et al., 2003, 2011; Smits & Monden, 2009; Vaupel et al., 2011; Wilmoth & Horiuchi, 1999; Wrycza et al., 2015):

1 $$\mathrm{AID}=\frac{1}{2}\sum_{x=0}^{x=\omega }\sum_{y=0}^{y=\omega }{d}_{x}{d}_{y}\left|\overline{x }-\overline{y }\right|,$$

2 $$G=\frac{\text{AID}}{{e}_{0}} .$$

In Eq. (1), $\overline{x },\overline{y }$ represent the mean age at death within the intervals $\left[x,x+1\right), [y,y+1)$. Equations (1) and (2) show the absolute and relative versions of the Gini index, respectively (Shkolnikov et al., 2003). The absolute Gini index, also known as ‘average interindividual difference’ (AID), measures the expected difference between ages at death when two individuals are picked randomly. The relative Gini index $G$ (also known as ‘Gini coefficient’) compares the AID with respect to the corresponding mean. It takes values between 0 and 1 (corresponding to the minimal and maximal inequality levels, respectively).

When presenting the empirical findings of the paper, we will be interested in showing what ages are mostly responsible for the observed changes in lifespan inequality. For that purpose, we will apply the decomposition method suggested by Horiuchi and colleagues that allows writing changes in lifespan inequality as the addition of age-specific components (see Horiuchi et al., 2008 for details).

## Normalized lifespan inequality

As hinted in the introduction, the fact that the mean of age-at-death distributions gradually approaches the maximal lifespan suggests that, since there is an increasingly smaller room for variation, lifespan inequality measures are mechanically forced to decline. In an attempt to adjust for such ‘boundary effect’, we propose a new approach to measure lifespan inequality. For any age-at-death distribution $A\in {D}^{\omega }$, we compare observed inequality levels $I\left(A\right)$ (where $I$ could be any inequality index) with respect to the ones that would be observed under a hypothetical distribution *with the same life expectancy* ${e}_{0}\left(A\right)$ that maximized $I$. This way, we derive a ‘relative-like’ measure that compares observed inequality levels against a mean-dependent benchmark case—thus facilitating comparisons of inequality levels between distributions with differing means.

In Appendix 1, we show that such mean-dependent inequality-maximizing age-at-death distribution is very simple to describe: it is a distribution where one portion of the population (with share ${s}_{1}$) dies at age 0 and the remaining population (with share ${1-s}_{1}$) dies at age $\omega$ (see some examples in Fig. 9). Such inequality-maximizing age-at-death distributions is denoted by $\mathcal{M}({e}_{0},\omega )$, where ${e}_{0}$ is the mean of the distribution. In a way, this mortality pattern is reminiscent of the hypothetical distribution that would be observed under an extreme rectangularization of a survival curve^4^ [see Fries (1980) for the statement of the rectangularization/compression hypothesis and Wilmoth and Horiuchi (1999) and Ebeling et al. (2018) for some suggestions on how to test and measure it]. Even if it is a hypothetical distribution that is unlikely to be observed in the real world, $\mathcal{M}({e}_{0},\omega )$ represents the benchmark case of extreme inequality against which we can compare length-of-life distributions. With such definitions, we now formally introduce our new class of inequality measures.

### **Definition**

Let *I* be a measure of inequality and $A\in {D}^{\omega }$ an age-at-death distribution. We define the corresponding *normalized inequality index* as:

3 $${I}^{*}\left(A,\omega \right):=\frac{I\left(A\right)}{I\left(\mathcal{M}({e}_{0}\left(A\right),\omega )\right)}.$$

By construction, I^*^ $(A,\omega )$ takes values between 0 and 1: it compares the observed inequality level $I\left(A\right)$ with the maximal value that $I$ could possibly take. It is easy to check that the values of ${I}^{*}$ can be interpreted *not only* as the ratio between observed inequality and the maximal inequality that could possibly exist, but *also* as a measure of the extent of lifespan variability. The normalization approach introduced in Eq. (3) can be applied to *any* inequality index. For the sake of simplicity, in this paper we restrict our attention to the absolute and relative Gini indices (see below). In Appendix 2, we show the normalized versions of popular inequality indicators that have been used in the analysis of lifespan variation, like the standard deviation ($\sigma$), the coefficient of variation ($\mathrm{CV}$), e-dagger (${e}^{\dagger}$) and the life table entropy ($H$).

### The absolute and relative Gini indices

Applying the previous definition to the average inter-individual difference (AID), we obtain the following normalized inequality index:

4 $${\mathrm{AID}}^{*}\left(A,\omega \right):=\frac{\mathrm{AID}(A)}{\left(\frac{{e}_{0}(A)(\omega -{e}_{0}(A))}{\omega }\right)}.$$

The denominator, i.e., maximal possible AID given ${e}_{0}(A)$ and $\omega$, equals ${e}_{0}(A)(\omega -{e}_{0}(A))/\omega$). Similarly, we can apply the normalization procedure to $G$ as follows (see proofs in Appendix 2):

5 $${G}^{*}\left(A,\omega \right):=\frac{G(A)}{\left(\frac{\omega -{e}_{0}(A)}{\omega }\right)}.$$

One can easily check that ${{\mathrm{AID}}^{*}\left(A,\omega \right)=G}^{*}\left(A,\omega \right)$. This means that the normalization procedure applied to the absolute and relative Gini indices yields the same inequality measure. Interestingly, this suggests that our normalization approach sidesteps the debated topic on whether length-of-life inequality should be calculated using absolute or relative measures. No matter if we start with one or the other, the normalization approach proposed in this paper brings together the absolute and relative worlds into a coherent whole.

### Sensitivity analysis

As can be seen in Eqs. (4–5), the normalized inequality indices depend on the value of $\omega$, the record human lifespan. The debate on the limits to human lifespan is long and inconclusive, so the choice of the value of $\omega$ is somewhat uncertain. Hence, we investigate the extent to which the inequality comparisons between pairs of length-of-life distributions ($A,B\in {D}^{\omega }$) are robust to alternative specifications of $\omega$. More specifically, we are interested in the following question: For what choices of $\omega$ is ${I}^{*}\left(A,\omega \right)$ greater than ${I}^{*}\left(B,\omega \right)$? When comparing ${I}^{*}\left(A,\omega \right)$ vis-à-vis ${I}^{*}\left(B,\omega \right)$, there are only two alternatives:

1. For all possible values of $\omega$, one of the two distributions is always deemed to be more or less unequal than the other (complete robustness).
2. There exists a *unique* threshold (denoted by ${\omega }^{*}$) above or below which one distribution is deemed more unequal than the other (partial robustness). The values of ${\omega }^{*}$ depend on the life expectancy and lifespan variability of $A$ and $B$ (details shown in Appendix 3).

This greatly simplifies the task of deciding whether the inequality ranking between $A$ and $B$ depends on the choice of $\omega$.

### Illustrative examples

**Fig. 2:**
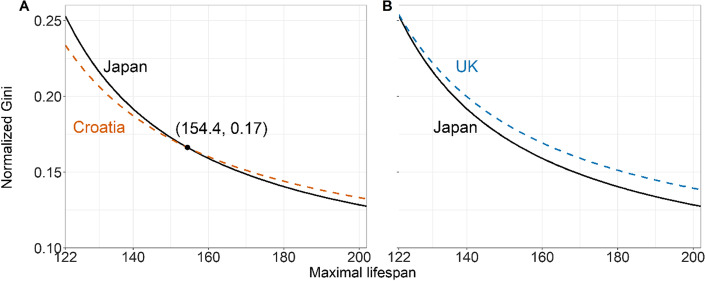
Normalized Gini with changing value of maximal lifespan (horizontal axis), Japanese females vs. Croatian females in 2015 (**A**), and Japanese females vs. UK females in 2015 (**B**) (Source: Authors’ elaboration based on the HMD data)

The robustness of different lifespan inequality comparisons is illustrated in the following examples using data from the HMD. Assume $A$ and $B$ correspond to the age-at-death distributions for Japanese and Croatian females in 2015, respectively. In the left panel of Fig. 2, we plot the values of ${G}^{*}\left(A,\omega \right)$ and ${G}^{*}\left(B,\omega \right)$ for values of $\omega$ above $122$ (the longest human lifespan ever recorded). The two curves have a single crossing point at ${\omega }^{*}=154.4$, where the ${G}^{*}$ is 0.17 for both Japanese and Croatian females. This means that when we choose values of $\omega$ between 122 and 154.4, Japanese females are more unequal than Croatian females by the normalized Gini, while the opposite conclusion is reached when $\omega$ is above 154.4. Thus, if there are compelling reasons to believe that maximal length of life is unlikely to exceed 154.4, we should conclude that lifespan inequality is lower among women in Croatia than in Japan.

Consider now a third distribution $C$ for UK women in 2015. It turns out that comparing the values of ${G}^{*}\left(A,\omega \right)$ and ${G}^{*}\left(C,\omega \right)$, the former is always lower than the latter, i.e., no matter what value of $\omega$ we choose, lifespan inequality is always higher among women in the UK than in Japan. This is illustrated in the right panel of Fig. 2, which shows that the two curves never cross when $\omega \ge 122$. This is an example of a ‘completely robust comparison’ among a pair of age-at-death distributions.

### Extreme uncertainty

What would happen if we were so uncertain about the value of the maximal lifespan $\omega$ that we allowed it to be indefinitely large? If one were not to impose any restriction whatsoever on human longevity, it turns out that when the maximal lifespan is allowed to be as large as possible, the normalized Gini index ${G}^{*}\left(A,\omega \right)$ converges to the classical Gini coefficient $G(A)$, so the latter can be seen as a particular case of the former (technical details shown in Appendix 4). This result nicely links the normalized inequality measures with the classical ones currently used in the literature, where no bounds are a priori imposed on the maximal human lifespan. Conversely, this also suggests that the Gini coefficient can be thought as a normalized inequality index, i.e., an index that compares observed inequality with respect to the maximal inequality one could possibly observe for a given mean.

## Revisiting the longevity–lifespan inequality nexus

In this section, we revisit lifespan inequality trends and their relationship with longevity investigated in previous studies (e.g., Edwards, 2011; Edwards & Tuljapurkar, 2005; Gillespie et al., 2014; Permanyer & Scholl, 2019; Smits & Monden, 2009; Vaupel et al., 2011) by introducing our normalized inequality measures to the debate. For that purpose, we use country-year specific period life tables from the HMD, which contains mortality data for 41 high-income countries over a long time span (some of them starting in the eighteenth century, but most of them starting somewhere in the twentieth century).

**Fig. 3:**
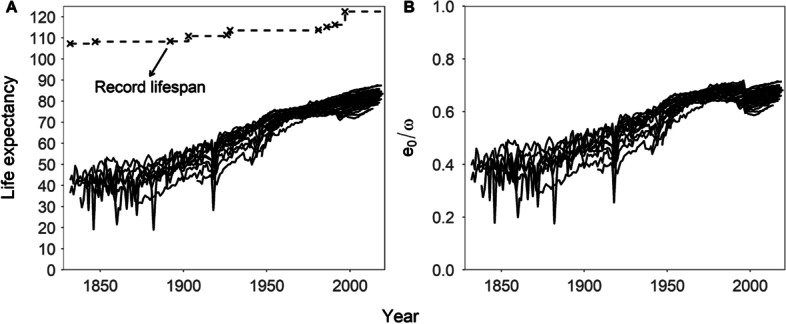
Trends of female life expectancy and record lifespan (**A**) and ratio between the two quantities (**B**), 1832–2020, HMD countries (Source: Authors’ elaboration based on the HMD data)

The left panel in Fig. 3 shows the trends in female life expectancy for the countries included in the HMD since 1832, the first year where human record lifespans were available. The complete series of record lifespans from the Gerontology Research Group (GRG) is also shown in the left panel of Fig. 3: it starts with the value of 107.26 in 1832 and gradually increases up to 122.45 (the age at death of Jeanne Calment) from 1997 onwards. As is well known, female life expectancy has increased over time for the countries included in the database. Despite occasional upheavals caused by wars, famines, epidemics or the failure of political systems, life expectancy has quickly resumed its vigorous upward trend. While both average and maximal longevity have increased over time, the rate of increase of the former has outpaced that of the later. As a result, the ratio between average and maximal longevity (${e}_{0}/\omega$) has generally increased, from 0.4 in the mid-nineteenth century up to 0.7 in recent years (see right panel of Fig. 3). As the average of the distribution approaches maximal length of life, there is less room for variability, so one of the expected consequences of the aforementioned trends should be a compression in the distribution of lifespans over time—an issue to which we now turn.

### Life expectancy against lifespan inequality

**Fig. 4:**
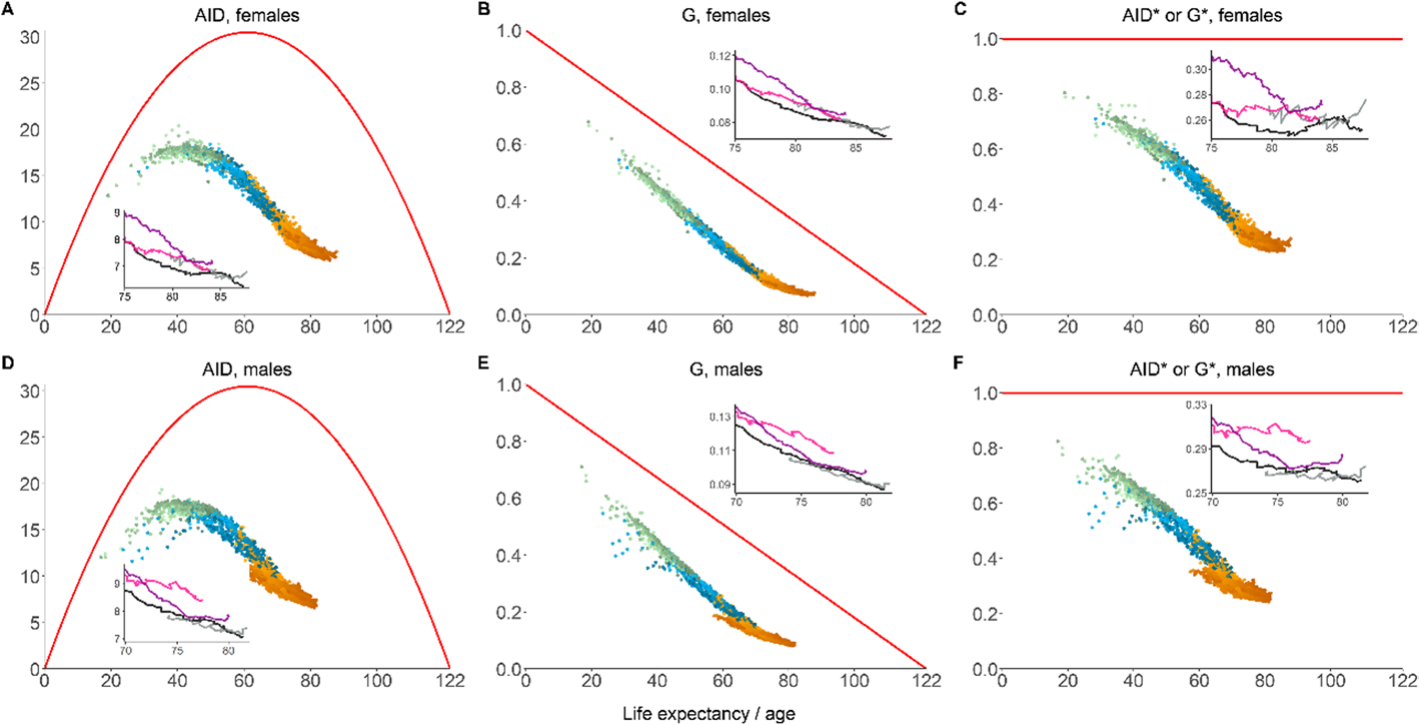
Life expectancy and lifespan inequality for different inequality indicators, 1832–2020, HMD countries. **A** Absolute Gini for females; **B** relative Gini for females; **C** normalized Gini for females; **D** absolute Gini for males; **E** relative Gini for males; **F** normalized Gini for males. Red curve for maximal inequality given maximal lifespan = 122, black line for Japan, dark magenta line for Canada, pink line for Taiwan, grey line for Hong Kong. Normalized indices were calculated when maximal lifespan equals 122. (Source: Authors’ elaboration based on the HMD data)

Panels A–F of Fig. 4 show the relationship between life expectancy and length-of-life inequality using several lifespan variability indicators (women in the upper row and men in the lower row). These scatterplots show, for all countries/regions and years included in the HMD, the levels of life expectancy in the horizontal axis and lifespan variability in the vertical one. The colors of the dots are used to indicate different time intervals (green for 1751–1899, blue for 1900–1949 and orange for 1950–2018), with darker shades indicating more recent years within each period. In each scatterplot, we superimpose a red curve indicating, for each level of life expectancy ${e}_{0}$, the maximal level of lifespan inequality we could possibly observe (i.e., the curve $I\left(\mathcal{M}({e}_{0},\omega )\right)$). In all cases, we choose $\omega =122$ (the oldest age-of-death ever recorded).^5^ Within each panel, we highlight the evolution over time for Japan, Canada, Taiwan and Hong Kong—four societies whose trajectories vary considerably across indicators.

The first column shows the results for the absolute Gini index (i.e., the AID). We observe an inverted U-shaped relationship between life expectancy and lifespan inequality. When longevity is ‘short’ (i.e., life expectancy around 30 years), the variability in length-of-life distributions is relatively high, but still not at its maximal height. As life expectancy increases, lifespan inequality increases until ${e}_{0}$ approaches 40 years. From that point onwards, lifespan inequality starts decreasing unabated. For Japan, Canada, Taiwan and Hong Kong, we observe a strong negative relationship between life expectancy and lifespan inequality, which becomes weaker when life expectancy goes beyond 80. The maximal lifespan inequality curve in panels A and D are an inverted parabola (the denominator in Eq. (4)), with a maximum when ${e}_{0}=\omega /2=61$ and then declining until ${e}_{0}$ reaches 122. This implies, when life expectancy approaches 122, everyone dies approximately at the maximum age and maximal inequality goes to zero.

The second column of Fig. 4 shows results for the relative Gini index. For these measures, the relationship between life expectancy and lifespan variability is monotonically decreasing and particularly strong, so there is less scope for ‘equality-efficiency’ trade-offs (i.e., increases in average longevity at the expense of higher variability). The clouds of points are very tight and from the values of longevity alone we can make very accurate guesses of the corresponding levels of lifespan inequality. In this case, the maximal inequality curve is a straight line (the denominator in Eq. (5)) that attains the value of zero when ${e}_{0}$ reaches 122. The highlighted trajectories of Japan, Canada, Taiwan and Hong Kong in the longevity–lifespan inequality space follow the same trend: increases in the former are almost invariably associated with decreases of the latter.

Lastly, the third column of Fig. 4 shows the results for the normalized inequality indices.^6^ For women and men, the associations roughly go in the same direction. When life expectancy ranges between 20 and 80, we observe a strong negative relationship between life expectancy and lifespan variability. When life expectancy exceeds 80, the relationship becomes flatter (i.e., further increases in longevity tend *not* to be accompanied by further decreases in lifespan variability), particularly for women. Indeed, for Japan, Canada, Taiwan and Hong Kong, we observe the emergence of plateaus and even some trend reversals (i.e., *increases* in longevity come with *increases* in lifespan variability). The fact that ${G}^{*}$ decreases with increasing longevity when ${e}_{0}$ ranges between 20 and 80 indicates that the rates of decline of $\mathrm{AID}$ and $G$ have outpaced the rate of decline of the corresponding maximal inequality curves (see panels A–E). Beyond the threshold ${e}_{0}\approx 80$, the decline of $\mathrm{AID}$ and $G$ slows down and cannot keep pace with the decline of the corresponding maximal inequality curves, thus leading to the leveling-off observed at the bottom-right corner of panels C and F (particularly for women).

### Correlation over time

**Fig. 5:**
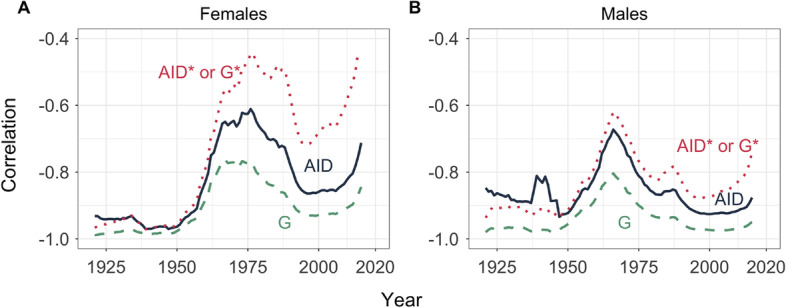
Pearson correlation between life expectancy and lifespan inequality measures (absolute, relative and normalized Gini indices) over different periods. **A** Results for females; **B** results for males. Calculations are based on HMD country-year observations from year $T-2$ to $T+2$ (Source: Authors’ elaboration based on the HMD data)

The results in Fig. 4 suggest that, as we focus our attention on recent periods, the relationship between longevity and lifespan inequality might become weaker. This is examined in the different panels of Fig. 5, where we plot the Pearson correlation coefficients between life expectancy and lifespan variability for the absolute, relative and normalized Gini indices (women in the left panel and men in the right panel). More specifically, we show the results corresponding to 5-year observation windows centered around year *T* (shown in the horizontal axes, i.e., observations included in years $T-2,T-1,T,T+1$ and $T+2$). Put differently, we plot the short-run relationships between life expectancy and lifespan inequality over time. The patterns are similar in both panels; the relationship between life expectancy and lifespan inequality is strongest for relative measures (negative and hovering around $-0.9$), and weaker for absolute and, especially, normalized measures (correlations closer to zero, yet negative). The hump that can be identified in both panels around the 1960–1980 period is partly attributable to the incorporation of Eastern and Central European countries to the HMD around the 1960s—which, at that time, had mortality patterns differing considerably with respect to the countries already included in the database. Yet, the rebound in the correlation coefficients observed since the turn of the century (particularly strong for women, see panel A) cannot be attributable to the inclusion of further countries to the database,^7^ but rather to a weakening relationship between life expectancy and lifespan inequality in recent times.

**Fig. 6:**
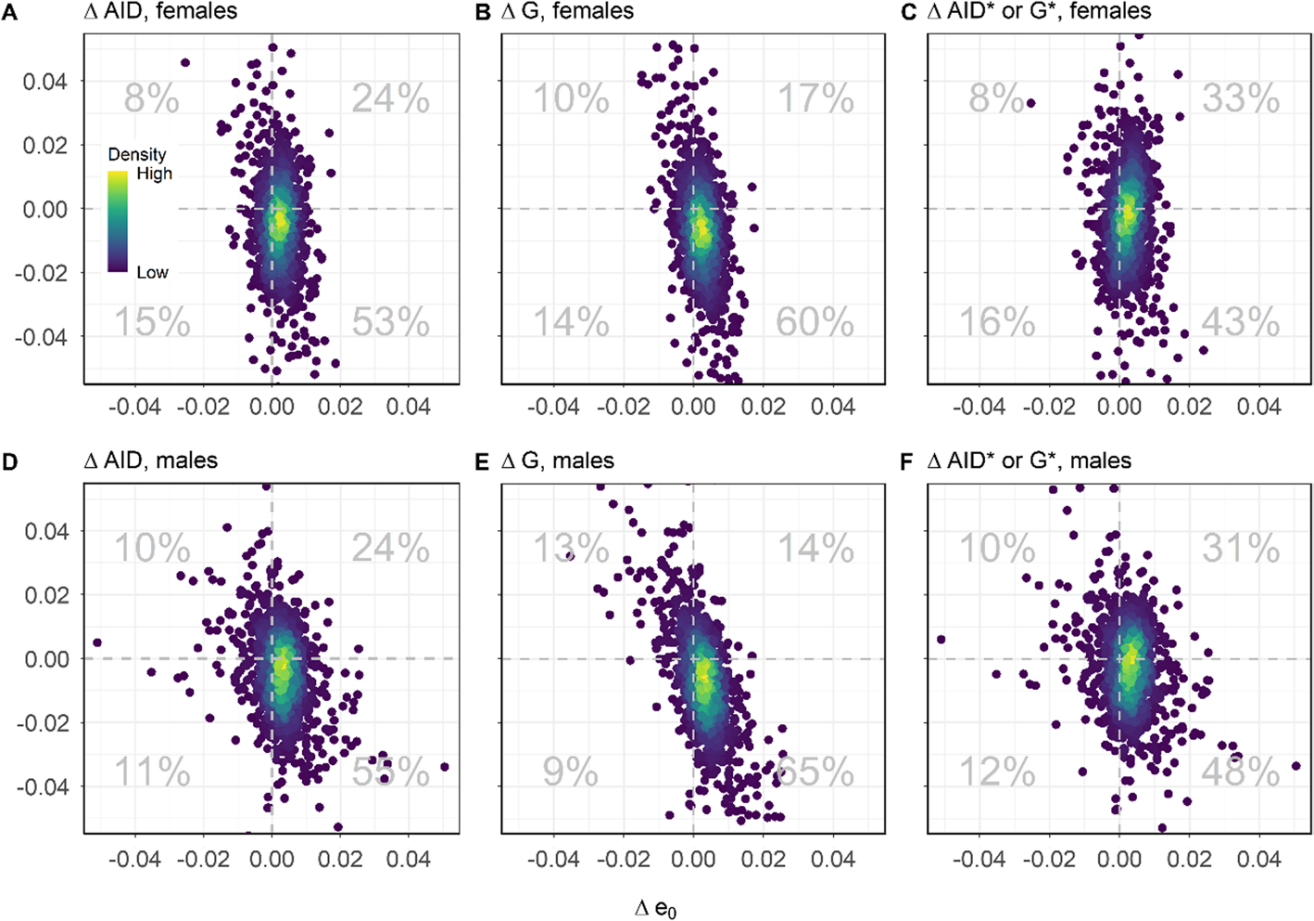
Joint changes in life expectancy and lifespan variability for the average inter-individual difference (**A**, **D**), Gini coefficient (**B**, **E**), normalized Gini index (**C**, **F**) from 1980 onwards. The top and bottom panels show results for women and men, respectively. Percentages based on the number of occurrences in each quadrant (Source: Authors’ elaboration based on the HMD data)

On their own, the shapes of the scatterplots shown in Fig. 4 do not tell us anything about the simultaneous direction of change. To measure the *joint* variability patterns of life expectancy and lifespan inequality, panels in Fig. 6 plot the relative changes of both variables (between every 2 consecutive years) against each other, including all possible changes from 1980 onwards—the period when, according to Fig. 5, the relationship between both variables becomes weaker. The shares of changes where life expectancy and lifespan inequality move simultaneously in the normatively desirable direction (i.e., increasing for life expectancy and declining for lifespan inequality) varies across indicators. While they are as high as 60% and 66% for the relative Gini index for women and men (see the lower right quadrants in the second-column panels), they hover around 53% and 55% for the absolute Gini for women and men (see first-column panels), and are appreciably lower for the normalized measures. For the normalized Gini index, life expectancy and lifespan inequality only move in the ‘right’ direction 43% and 48% of the times for women and men. On many occasions, both variables move in directions ‘benefiting’ one to the detriment of the other (i.e., simultaneous increases (or decreases) in both life expectancy *and* lifespan inequality). The upper right and lower left quadrants in the different panels of Fig. 6 show that the normatively undesirable changes happen around 35–38% of the times for the absolute Gini index, around 22–30% for the relative Gini, and 43–49% for the normalized Gini.

### Age decomposition patterns

**Fig. 7:**
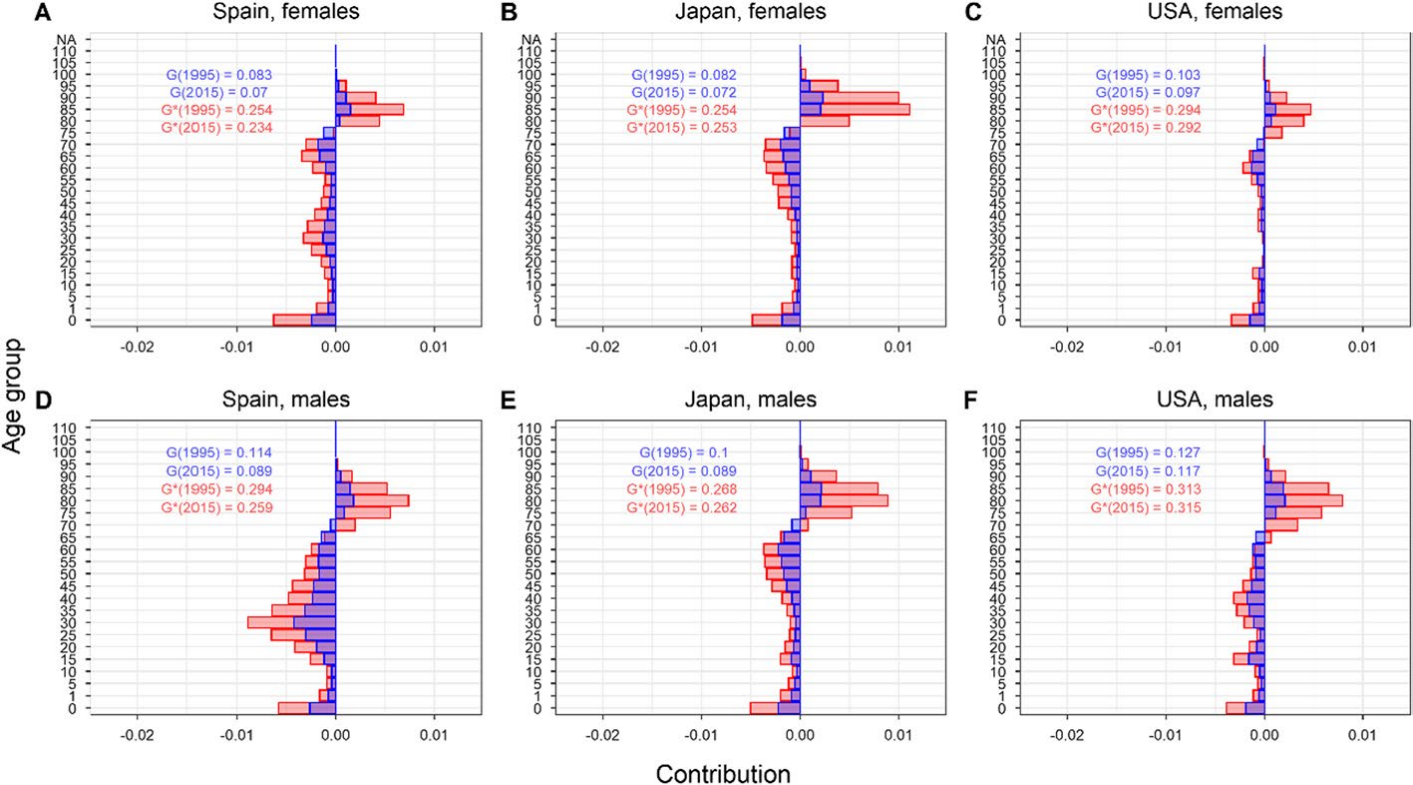
Age-specific decompositions of lifespan inequality change for females (top row) and males (bottom row) in Spain, Japan, and the US between 1995 and 2015. The blue bars show the age-specific contributions for the Gini index and the red ones for the normalized Gini index (Source: Authors’ elaboration based on the HMD data)

In Fig. 7, we show the contribution of each age group to changes in the Gini index (blue bars) and the normalized Gini index (red bars) between 1995 and 2015 for females (top row) and males (bottom row) in the US, Spain (the countries with highest and lowest relative Gini index in 2015 in the database, respectively) and Japan (the country with the highest life expectancy in 2018). The results for other HMD countries are similar, and are shown in Figs. 12, 13 in Appendix 5. Irrespective of the chosen inequality measure, there is a clear pattern, whereby mortality declines at older ages contribute to increasing lifespan inequality, while the opposite happens at younger ages. The age separating ‘early’ from ‘late’ deaths is referred to as the ‘threshold age’ (Aburto et al., 2019; Gillespie et al., 2014; Zhang & Vaupel, 2009). As can be inferred from the direction of the bars, the threshold age is similar—yet not exactly the same—for the Gini and the normalized Gini indices.

For the three countries shown in Fig. 7, the overall negative contributions at younger ages are larger than the overall positive contributions at older ages, thus resulting in an overall decline in lifespan inequality, both for the Gini index and its normalized version. Interestingly, the age-specific contributions tend to be substantially larger for the normalized Gini in both directions (i.e., red bars are substantially longer than blue bars), particularly at advanced ages. Inspecting the relative size of the bars, it is clear that the Gini index is less sensitive to changes occurring at older ages than its normalized counterpart. Indeed, the inequality-enhancing contributions occurring at those older ages is so large that the normalized Gini index barely declines for the US and Japan, while it declines considerably for the standard Gini index (a pattern that is observed in several other HMD countries, see Figs. 12, 13 in Appendix 5).

### Sensitivity to the choice of $\omega$

**Fig. 8:**
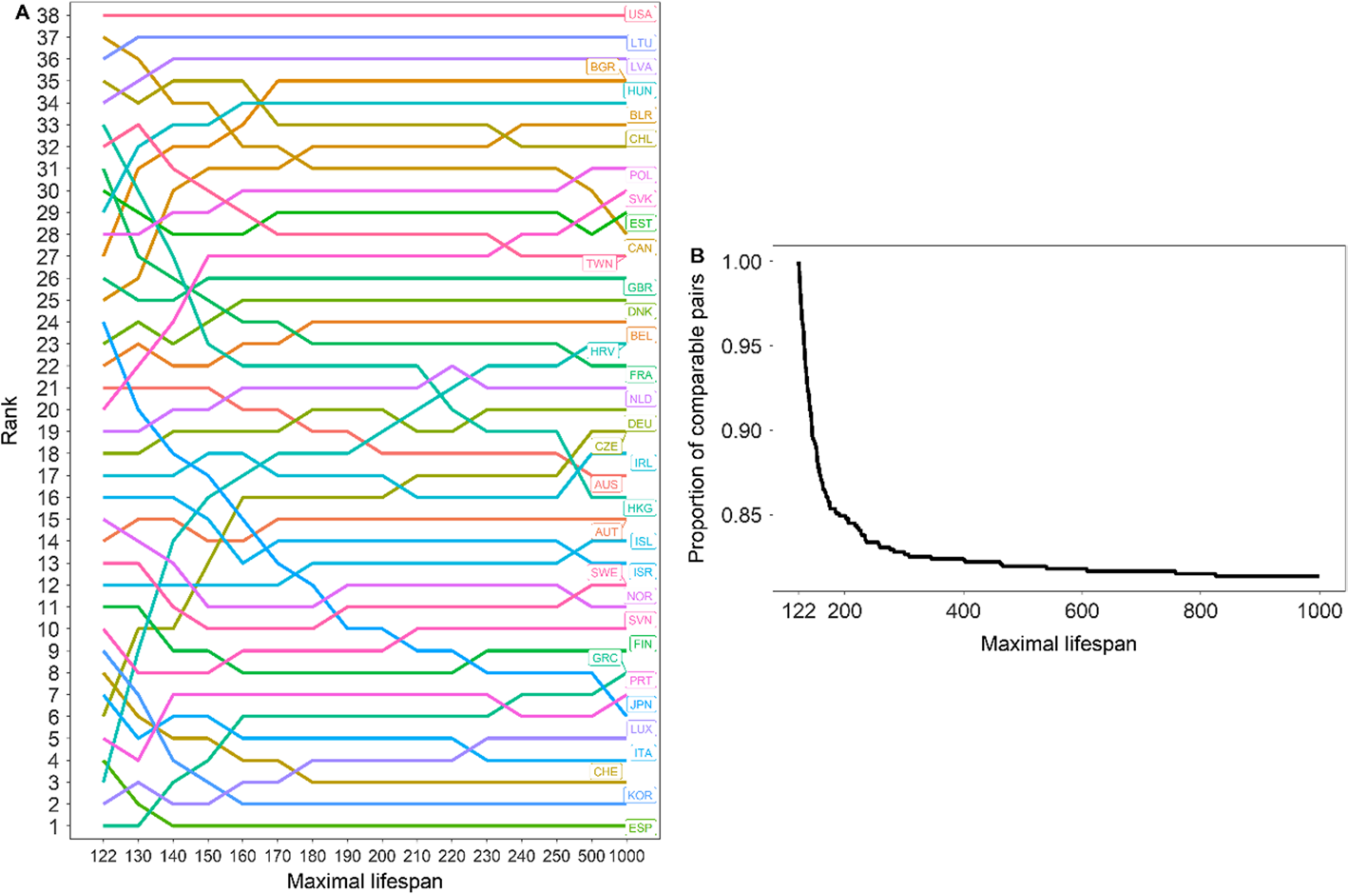
Country rank (**A**) and proportion of comparable pairs (**B**) by maximal lifespan (horizontal axis), females, 2015 (Source: Authors’ elaboration based on the HMD data)

**Fig. 9:**
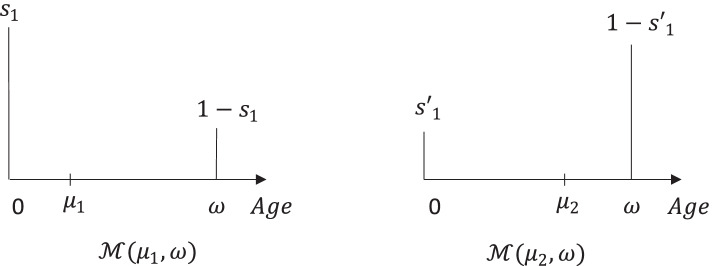
Two generic examples of inequality-maximizing distributions for different mean values (${\mu }_{1}$ and ${\mu }_{2}$). (Source: Authors’ elaboration)

All the findings reported so far crucially depend on the choice of the maximum reported age at death. Since this value has increased over time and is likely to continue increasing in the foreseeable future, it is important to investigate the sensitivity of our findings to alternative specifications of $\omega$—other than 122. The left panel of Fig. 8 shows the ranking of the 32 HMD countries/regions included in 2015 according to the values of the normalized Gini index applied to female age-at-death distributions when $\omega$ ranges between 122 (the lowest possible value it could take) and 1000 (an unreasonably large number that safely includes most of the reasonable upper bounds one could possibly choose). When $\omega =1000$, ${G}^{*}$ is essentially the same as $G$. Lower ranking values (i.e., near ‘1’) indicate a lower level of lifespan inequality (as measured by ${G}^{*}$). Generally speaking, when $\omega$ is near 122, the populations with shorter life expectancies (e.g., Eastern European countries, such as Croatia, the Czech Republic, Belarus, Bulgaria or Slovakia) tend to fare better with the normalized Gini index than those at the other extreme of the longevity distribution (e.g., Japan, Hong Kong, France). Very often, this can be explained by the fact that the normalize Gini index is the same as the Gini index times a ‘correcting factor’ (equal to $\omega /(\omega -{e}_{0})$; see Eq. (5)) that, other factors kept constant, benefits countries with shorter life expectancies (see Panel A in Fig. 2). Yet, there are notable exceptions to such general patterns. For example, Spain has a high level of life expectancy *and* a very low level of lifespan inequality in terms of ${G}^{*}$, no matter what value of $\omega$ we choose. Symmetrically, Lithuania, Latvia and, most notably, the US, have relatively low life expectancies, but nevertheless exhibit relatively high levels of ${G}^{*}$ for all values of $\omega$.

The left panel of Fig. 8 shows that, most of the country reshuffling takes place when $\omega$ ranges between 122 and, say, 160. Beyond 160, the country ranking remains relatively stable. This is also illustrated in the right panel of Fig. 8, which plots the proportion of country pairs that are consistently ranked for all possible values of $\omega$ included between 122 and the specific value in the horizontal axis. To illustrate: 83% of all possible country pairs can be consistently ranked no matter what value of $\omega$ we choose between 122 and 250. When the range of admissible values of $\omega$ increases, the proportion of countries that can be consistently ranked by all of them naturally decreases. The curve depicted in the right panel of Fig. 8 declines steeply when the maximal lifespan moves between 122 and 160 (the range of values where most of the country re-ranking takes place), but gradually stabilizes for higher values of $\omega$. In the limit, it turns out that 81% of all country pairs can be consistently ranked when $\omega$ ranges between 122 and 1000. Thus, the country rankings arising from the values of ${G}^{*}$ and $G$ are relatively similar, but important differences can be identified when the values of maximal lifespan are assumed to be below 160.

## Discussion and concluding remarks

### Summary of the findings

In recent years, several studies have documented a strong negative association between longevity and lifespan variability (Colchero et al., 2016; Edwards, 2011; Smits & Monden, 2009; Vaupel et al., 2011). We showed that as life expectancy increases at a faster pace than the maximal human lifespan, age-at-death distributions are mechanically compressed into a gradually narrower age range, eventually leading to a decrease in lifespan inequality. To probe further into this important issue, we have proposed a new class of measures: the ‘normalized lifespan inequality’ indices. This new class of lifespan inequality measures explicitly takes into consideration the reduced room for variation that ensues when life expectancy approaches the maximal human lifespan. Interestingly, the suggested normalization approach gives the same results for absolute and relative inequality measures, thus sidestepping a long and inconclusive debate.

**Fig. 10:**
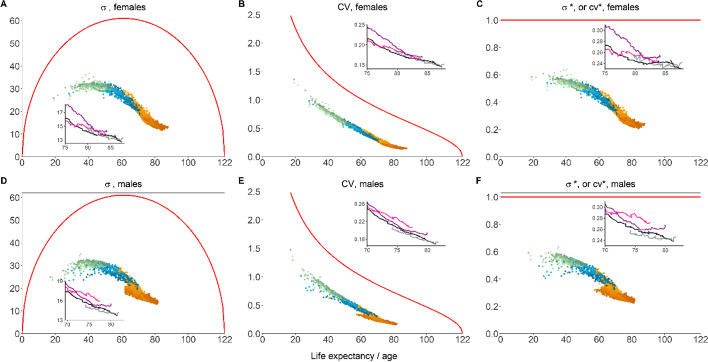
Life expectancy and lifespan inequality for different inequality indicators, 1832–2020, HMD countries. **A** Standard deviation for females; **B** coefficient of variation for females; **C** normalized standard deviation or coefficient of variation for females; **D** standard deviation for males; **E** coefficient of variation for males; **F** normalized standard deviation or coefficient of variation for males. Red curve for maximal inequality given maximal lifespan = 122, black line for Japan, dark magenta line for Canada, pink line for Taiwan, grey line for Hong Kong. Normalized indices were calculated when maximal lifespan equals 122 (Source: Authors’ elaboration based on the HMD data)

**Fig. 11:**
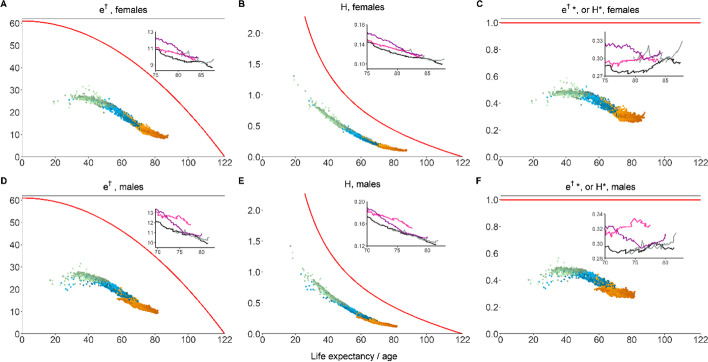
Life expectancy and lifespan inequality for different inequality indicators, 1832–2020, HMD countries. **A** Life disparity for females; **B** life table entropy for females; **C** normalized life disparity or life table entropy for females; **D** life disparity for males; **E** life table entropy for males; **F** normalized life disparity or life table entropy for males. Red curve for maximal inequality given maximal lifespan = 122, black line for Japan, dark magenta line for Canada, pink line for Taiwan, grey line for Hong Kong. Normalized indices were calculated when maximal lifespan equals 122. (Source: Authors’ elaboration based on the HMD data)

**Fig. 12:**
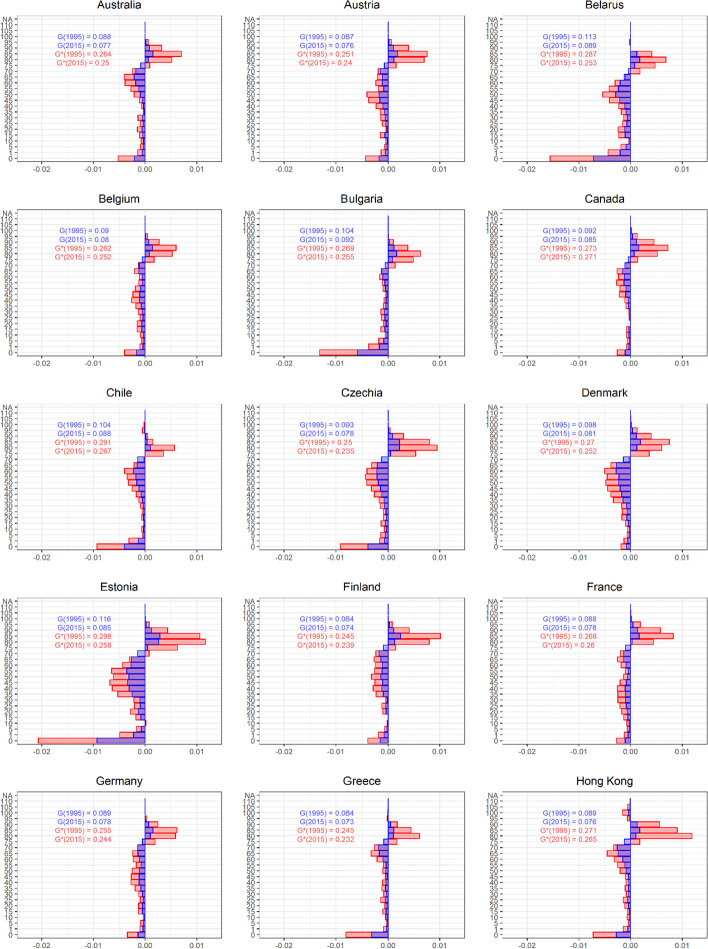

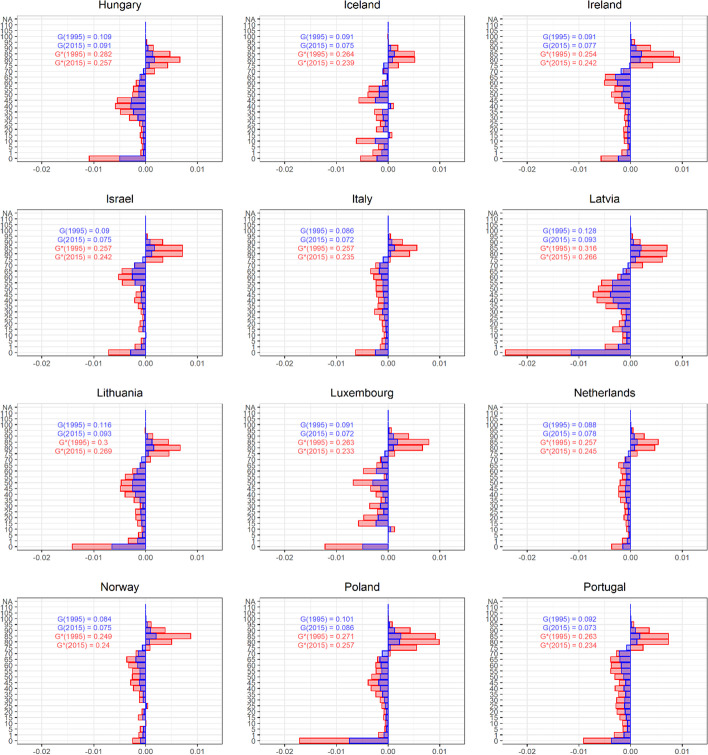
Age-specific decompositions of lifespan inequality change for females between 1995 and 2015 in HMD countries. The blue bars show the age-specific contributions for the Gini index and the red ones for the normalized Gini index (Source: Authors’ elaboration based on the HMD data)

**Fig. 13:**
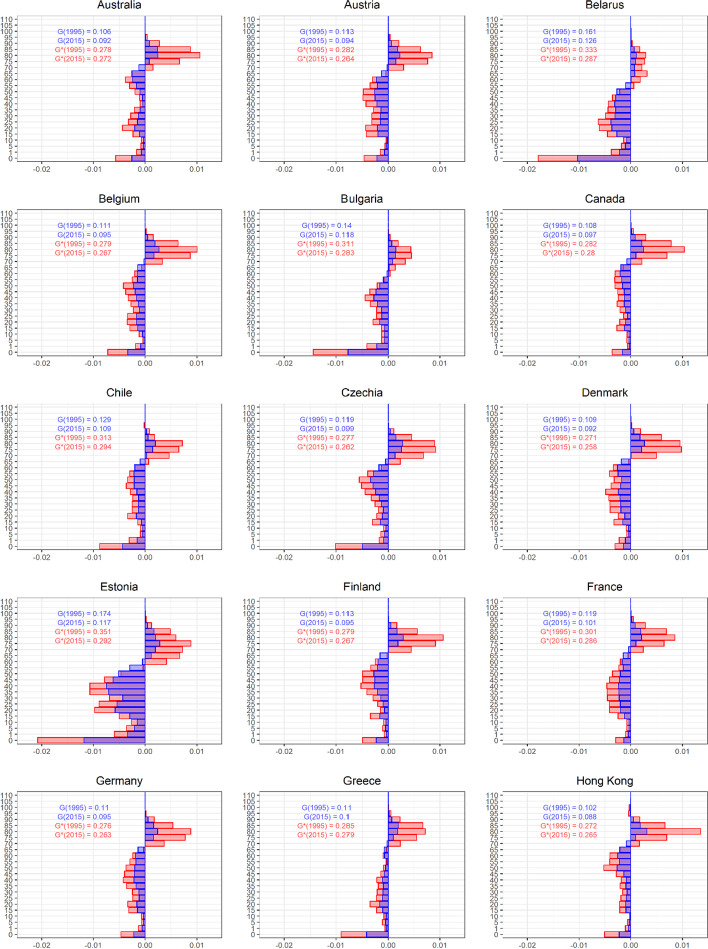

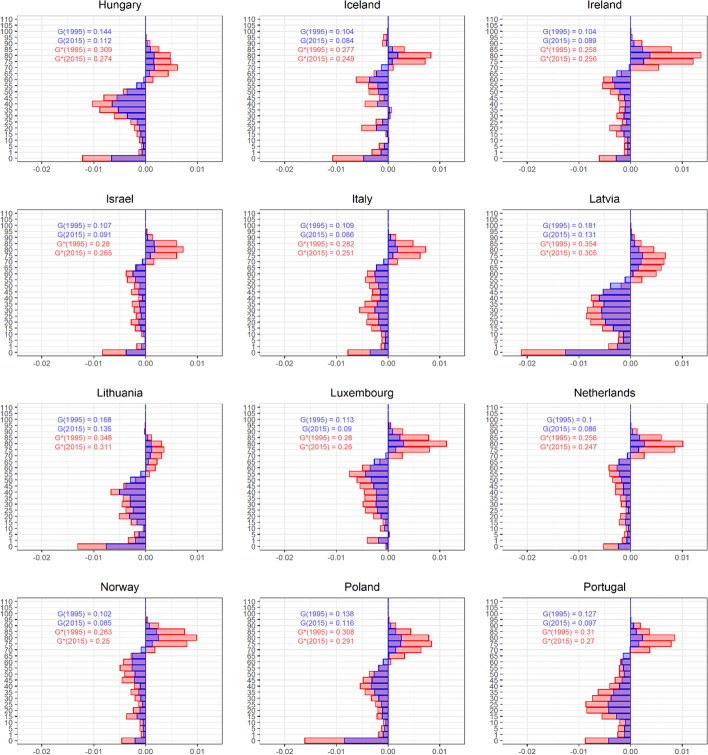
Age-specific decompositions of lifespan inequality change for males between 1995 and 2015 in HMD countries. The blue bars show the age-specific contributions for the Gini index and the red ones for the normalized Gini index (Source: Authors’ elaboration based on the HMD data)

What can researchers and practitioners learn from our approach? The normalized inequality indices provide analysts who are willing to make assumptions on a certain maximum lifespan with an opportunity to quantify maximum lifespan-adjusted inequality. This gives additional insights that conventional indices miss. Absolute measures of lifespan inequality assess the spread of length-of-life distributions and are often measured in ‘number of years’ (i.e., their values are reported using the same measurement unit as the original variable). In order to render distributions with different means more comparable, relative measures of inequality typically divide the level of absolute inequality by the corresponding mean. Yet, when the variable (age-at-death) has an uncertain upper bound ($\omega$), accounting for the values of mean alone might not be enough. Indeed, when the mean approaches such upper bound, absolute and relative inequalities mechanically decline—a circumstance that complicates the inequality comparison of distributions with different means, which does not affect the measurement of inequality of unbounded variables (e.g., income). The normalized inequality measures address such issues. In addition, our approach can be implemented to any inequality metric, such as the standard deviation or the life table entropy (see equations in Appendix 2 and results in Figs. 10, 11 in Appendix 5). The new indicators are thus a useful complement to practitioners’ toolkit of mortality analysis.

By incorporating the three components simultaneously (absolute inequality, mean, and maximal lifespan), the proposed measures offer additional insights to the conceptualization of lifespan inequality. Inter alia, they can be very useful to bio-demographers and zoologists comparing longevity and lifespan variability across species.^8^ In addition, the new measures can inform public health policies—which over the last years have gone beyond the promotion of long and healthy lives to incorporate equity concerns, e.g., ensuring that increasing longevity benefits *all* socio-economic groups (Benach et al., 2011, 2013; Brønnum-Hansen, 2017). The use of normalized measures facilitates the comparison of lifespan inequality across countries or social groups with different longevity levels.

Empirically, we have investigated how the normalized inequality indices behave using the HMD. It is remarkable that, even after controlling for the shrinking space for variability stemming from the increasing proximity between ${e}_{0}$ and $\omega$, we still observe a strong negative relationship between normalized lifespan inequality and ${e}_{0}$ when the latter is below 80 years. Beyond that threshold, instead of converging to zero (as the extreme version of Fries’ (1980) compression-rectangularization hypothesis would predict), normalized lifespan inequality seem to reach a previously unobserved plateau well above zero. Indeed, for a certain group of countries/regions we even observe trend *reversals*, that is: at high longevity levels, further increases in life expectancy go in tandem with increases in lifespan inequality (for women in Japan, Hong Kong, and, to a lesser extent, Canada). Even if the relationship between longevity and lifespan inequality has traditionally been strong and negative in a long-run perspective (e.g., from the nineteenth century onwards), our findings point to the emergence of a length-of-life inequality plateau at higher longevity levels. Thus, we might be entering a new period where further increases in longevity might not be necessarily accompanied by reductions of lifespan inequality.

### Comparison with other studies

The plateauing and reversal of lifespan variability as longevity increases is reminiscent of the findings from several prior studies (Cheung & Robine, 2007; Engelman et al., 2010; Permanyer & Scholl, 2019), which find that length-of-life inequality is stagnating or increasing *among older adults*. The normalized inequality indices show that lifespan variability *for the entire population* that might also rise at higher longevity levels. Indeed, recent studies in high-income countries indicate that the causes of death that contributed most to declines in the variance are different from those that contributed most to increase in life expectancy (Seligman et al., 2016), an issue that might generate trade-offs between raising average longevity and reducing variation. Similarly, Aburto and Van Raalte (2018) identify several instances in Central and Eastern Europe where the desirable goal of increasing longevity is at odds with the goal of reducing length-of-life inequality. If health improvements contribute to increase longevity *and* lifespan variability simultaneously, healthcare systems may face an ethical dilemma.

A recent strand of research has investigated the relationship between life expectancy and lifespan variability by showing the threshold age separating ‘early’ from ‘late’ deaths, and saving lives above the threshold increases lifespan inequality (Aburto et al., 2019; Gillespie et al., 2014; Zhang & Vaupel, 2009)—a pattern we have also observed in our normalized inequality measures (Fig. 7). As long as mortality improvements below the threshold age outpace those above it, life expectancy will increase while lifespan inequality decreases (Aburto et al., 2019). Since such a threshold age increases over time, these studies do not expect the association between life expectancy and lifespan inequality to be reversed. A fundamental difference between the ‘threshold age’ approach and the one presented here is the assumptions regarding the maximal lifespan. The aforementioned studies assume that $\omega =+\infty$, whereas we consider that $\omega$ is finite.

Given the uncertainty surrounding the values of $\omega$, we have (i) allowed them to increase over time as new maximal lifespan records are achieved; (ii) showed how to check the robustness of our findings to alternative values of $\omega$, and (iii) investigated the implications of letting $\omega$ to be indefinitely large. As discussed in the third section, when $\omega$ is allowed to increase indefinitely,^9^ our normalized Gini index converges towards the traditional Gini index, bridging the bounded and unbounded worlds into a coherent whole. Future research could attempt to unify both ‘threshold age and $\omega <+\infty$ approaches’ into a single, all-encompassing analytical strategy.

We are not the first to account for the ‘boundary effect’ that is generated when life expectancy approaches the maximal lifespan. Smits and Monden (2009) proposed the ‘Relative Length of Life Inequality’ index (RLI), which is calculated by standardizing the length-of-life inequality scores within 1-year ranges of life expectancy. Thus, it represents the deviation from average length-of-life inequality at a certain level of life expectancy in units of one standard deviation. While this solves the problem of mean-dependency, it generates other difficulties. The RLI($A$) of a given country depends not only on the corresponding age-at-death distribution ($A$), but also on the age-at-death distributions of *all other* countries having a life expectancy *included* in the interval $[{e}_{0}\left(A\right)-1,{e}_{0}\left(A\right)+1]$. Further, which countries are included also affects the results. This not only complicates the interpretation of the index, but also compromises its comparability over time. While the RLI can be said to be ‘relative’ (as it depends on other countries’ length-of-life distributions), our normalization can be said to be ‘absolute’ (as it is unaffected by other countries’ age-at-death distributions). The use of the same inequality benchmark ($I\left(\mathcal{M}({e}_{0},\omega )\right)$) for all distributions having the same mean ${e}_{0}$ is an advantage of our approach.

### Future prospects

What can we learn about the prospects of the relationship between longevity and normalized lifespan inequality? As discussed earlier, one of the key factors determining such relationship is the relative magnitude of life expectancy vis-à-vis maximal length of life. Assuming both ${e}_{0}$ and $\omega$ continue to increase over time (as presumed by most current projections), the speed at which the two magnitudes increase will be one of the key determinants. If ${e}_{0}$ continues to increase faster than $\omega$ does, then we should expect further decreases in normalized lifespan inequality. Yet, it is unclear whether this will continue to be the case in the coming decades. Indeed, Panel B in Fig. 3 suggests that the ratio between average and maximal record longevity has stagnated in the last decades. For one thing, the pace of life expectancy gains has slowed down since the 1950s, both globally and in high-income countries (Cardona & Bishai, 2018; Leon et al., 2019). For another, there are growing health differences within countries across socio-economic lines,^10^ a factor that could potentially hinder prospective improvements in countries’ life expectancy. On top of this, the outbreak of the Covid-19 pandemic is an extreme shock that shortens life expectancy, reduces lifespan inequality (as older adults are the most vulnerable), and exacerbates socioeconomic differences in longevity (Aburto et al., 2021; Chen & Krieger, 2020). Lastly, the increasing survival of supercentenarians observed during the last decades might soon push maximal length of life to unprecedentedly high levels (Maier et al., 2010; Medford & Vaupel, 2019; Zuo et al., 2018). If these different forces continue operating simultaneously for a sufficiently long time, we might increasingly observe reversals in the relationship between longevity and normalized lifespan inequality. If so, it might be increasingly difficult to simultaneously achieve the normatively desirable goals of increasing longevity *and* reducing length-of-life inequality.

## Data Availability

All the data used in this paper come from the Human Mortality Database (https://www.mortality.org/), which is freely accessible to researchers all over the world.

## Appendix 1

In order to operationalize the ‘normalized inequality approach’, one has to define what is the hypothetical distribution (with a given mean ${e}_{0}$) maximizing any inequality index. The following proposition characterizes such inequality-maximizing age-at-death distributions.

### **Proposition**

*Let* ${I:D}^{\omega }\to {\mathbb{R}}_{+}$ *be an inequality index. For a given mean* ${e}_{0}\in [0,\omega ]$, the age-at-death distribution with mean ${e}_{0}$ maximizing $I$ is a distribution where the population is split in two groups: the first one with a share ${s}_{1}$ attaining a value of zero and the other one (with a share of $1-{s}_{1}$) attaining $\omega$, in such a way that ${e}_{0}={s}_{1}\cdot 0+\left(1-{s}_{1}\right)\cdot \omega$. Such inequality-maximizing distribution will be denoted as $\mathcal{M}({e}_{0},\omega )$.

### Proof of proposition

This result is an immediate consequence of Theorem 1 in Seth and Yalonetzky (2016). According to that result, the Lorenz curve of the distribution $\mathcal{M}({e}_{0},\omega )$ will always be below the Lorenz curve of any other age-at-death distribution in $[0,\omega ]$ with mean ${e}_{0}$. This implies that all inequality indices satisfying the Pigou–Dalton Transfer Principle and the properties of Symmetry and Minimal Inequality^11^ will deem $\mathcal{M}({e}_{0},\omega )$ more unequal than any other distribution in $[0,\omega ]$ with mean ${e}_{0}$, as we wanted to demonstrate.^12^

Thus, such inequality-maximizing age-at-death distribution is very easy to describe: it is a bipolar distribution where one portion of the population (with share ${s}_{1}$) dies at age 0 and the remaining population (with share ${1-s}_{1}$) dies at age $\omega$. In Fig. 9, we illustrate how such distributions look like for a couple of generic values of ${e}_{0}$ (denoted as ${\mu }_{1}$ and ${\mu }_{2}$). As the mean ${e}_{0}$ approaches $\omega$, the share of the group dying at age 0 (*s*_1_) gradually goes to zero.

## Appendix 2

In this appendix, we derive the values of the different inequality measures presented in this paper for the inequality-maximizing distributions $\mathcal{M}({e}_{0},\omega )$ described in Proposition 1. In all cases, we use the fact that, because of the way in which such distributions have been defined

A1 $${e}_{0}={s}_{1}\cdot 0+\left(1-{s}_{1}\right)\cdot \omega =\left(1-{s}_{1}\right)\cdot \omega .$$

### The average inter-individual difference

Applying Eq. (1) to the inequality-maximizing distribution $\mathcal{M}({e}_{0},\omega )$ described in Appendix 1, one has that

A2 $$\mathrm{AID}\left(\mathcal{M}({e}_{0},\omega ) \right)=\frac{2{s}_{1}(1-{s}_{1})\omega }{2}.$$

Plugging (A1) into (A2) and manipulating algebraically, we have that

A3 $$\mathrm{AID}\left(\mathcal{M}({e}_{0},\omega ) \right)={s}_{1}\left(1-{s}_{1}\right)\omega =\frac{\frac{{e}_{0}}{\omega }\left(1-\frac{{e}_{0}}{\omega }\right)\omega {=e}_{0}\left(\omega -{e}_{0}\right)}{\omega }.$$

### The Gini coefficient

Applying Eq. (2) to the inequality-maximizing distribution $\mathcal{M}({e}_{0},\omega )$ described in Appendix 1, one has that

A4 $$G\left(\mathcal{M}({e}_{0},\omega )\right)=\frac{{s}_{1}(1-{s}_{1})\omega }{{e}_{0}}.$$

Plugging (A1) into (A4) and manipulating algebraically, we have that

A5 $$G\left(\mathcal{M}({e}_{0},\omega )\right)=\frac{{e}_{0}(\omega -{e}_{0})/\omega }{{e}_{0}}=\frac{\omega -{e}_{0}}{\omega }.$$

### The standard deviation

The standard deviation, $\sigma$, is given calculated as

A6 $$\sigma =\sqrt{\sum_{x=0}^{x=\omega }{d}_{x}{\left(\overline{x }-{e}_{0}\right)}^{2}} .$$

Applying Eq. (A6) to the inequality-maximizing distribution $\mathcal{M}({e}_{0},\omega )$ described in Proposition 1, one has that

A7 $$\sigma \left(\mathcal{M}({e}_{0},\omega )\right)=\sqrt{{s}_{1}{e}_{0}^{2}+(1-{s}_{1}){\left(\omega -{e}_{0}\right)}^{2}} .$$

Plugging (A1) into (A7), we have that

A8 $$\sigma \left(\mathcal{M}({e}_{0},\omega )\right)=\sqrt{\left(1-\frac{{e}_{0}}{\omega }\right){e}_{0}^{2}+\frac{{e}_{0}}{\omega }{\left(\omega -{e}_{0}\right)}^{2}} .$$

Manipulating algebraically, we conclude that

A9 $$\sigma \left(\mathcal{M}({e}_{0},\omega )\right)=\sqrt{{e}_{0}\left(\omega -{e}_{0}\right)}$$

### The coefficient of variation

The coefficient of variation, $\mathrm{CV}$, is calculated as

A10 $$\mathrm{CV}=\frac{\sigma }{{e}_{0}}.$$

Applying Eq. (A10) to the inequality-maximizing distribution $\mathcal{M}({e}_{0},\omega )$ described in Proposition 1, one has that

A11 $$\mathrm{CV}\left(\mathcal{M}({e}_{0},\omega )\right)=\frac{\sqrt{{s}_{1}{e}_{0}^{2}+(1-{s}_{1}){\left(\omega -{e}_{0}\right)}^{2}}}{{e}_{0}}.$$

Plugging (A1) into (A11) and manipulating algebraically, we have that

A12 $$CV\left(\mathcal{M}({e}_{0},\omega )\right)=\frac{\sqrt{{e}_{0}\left(\omega -{e}_{0}\right)}}{{e}_{0}}=\sqrt{\frac{\omega -{e}_{0}}{{e}_{0}}} .$$

### Life disparity (e-dagger)

Life disparity, e-dagger or ${e}^{\dagger}$, is calculated as follows:

A13 $${e}^{\dagger}=\sum_{x=0}^{x=\omega -1}{d}_{x}\left(\frac{{e}_{x}+{e}_{x+1}}{2}\right) .$$

Applying Eq. (A13) to the inequality-maximizing distribution $\mathcal{M}({e}_{0},\omega )$ described in Proposition 1, one has that

A14 $${e}^{\dagger}\left(\mathcal{M}({e}_{0},\omega ) \right)={s}_{1}\cdot \left(\frac{{e}_{0}+{e}_{1}}{2}\right)={s}_{1}\cdot \left(\frac{{e}_{0}+\omega }{2}\right).$$

Plugging (A1) into (A14) and manipulating algebraically, we have that

A15 $${e}^{\dagger}\left(\mathcal{M}\left({e}_{0},\omega \right)\right)=\frac{1}{2}\left(\left(1-\frac{{e}_{0}}{\omega }\right)\cdot \left({e}_{0}+\omega \right)\right)=\frac{{\omega }^{2}-{e}_{0}^{2}}{2\omega }.$$

### Life table entropy

The life table entropy, $H$, is given below:

A16 $$H=\frac{{e}^{\dagger}}{{e}_{0}}.$$

Applying Eq. (A16) to the inequality-maximizing distribution $\mathcal{M}({e}_{0},\omega )$ described in Proposition 1, one has that

A17 $$H\left(\mathcal{M}({e}_{0},\omega ) \right)=\frac{{s}_{1}\cdot \left({e}_{0}+{e}_{1}\right)}{2{e}_{0}}=\frac{{s}_{1}\cdot \left({e}_{0}+\omega \right)}{2{e}_{0}}.$$

Plugging (A1) into (A17) and manipulating algebraically, we have that

A18 $$H\left(\mathcal{M}\left({e}_{0},\omega \right)\right)=\frac{{\omega }^{2}-{e}_{0}^{2}}{2{e}_{0}\omega }.$$

## Appendix 3

Let $M$ be the longevity record prevailing at the time the distributions were estimated. In a setting where the choice of $\omega$ is uncertain, $M$ represents the lowest upper bound we can possibly choose. From now onwards, the half-bounded interval $\left[M,+\infty \right)$ will be referred to as ‘admissible’ or ‘feasible’ space. In order to investigate the robustness of the inequality ranking between $A$ and $B$, we have to identify the elements of the following set:

A19 $$S\left(A,B\right):=\left\{\omega \ge M|{{I}^{*}}\left(A,\omega \right)\le {I}^{*}\left(B,\omega \right)\right\},$$

i.e., the set of upper bounds for which $A$ is deemed less unequal than $B$. If it turns out that the set $S\left(A,B\right)$ contains all feasible upper bounds, we can safely conclude that $A$ has unambiguously less inequality than $B$. On the other hand, whenever $S\left(A,B\right)$ does not span across the entire admissible space, the inequality ranking between $A$ and $B$ depends on the choice of $\omega$. Ideally, when comparing the inequality of lifespan distributions one would like $S\left(A,B\right)$ to contain all feasible upper bounds to be sure that conclusions are completely robust. Yet, one should not automatically discard those comparisons failing to comply with the ‘complete robustness’ criterion. If it turns out that $S\left(A,B\right)=[M,U]$ with $U$ being an extremely high upper bound beyond which no human being is ever expected to survive (say, $U$ = 969: the purported age at death of Methuselah), one could quite confidently claim that $A$ exhibits less inequality than $B$.

In order to identify the elements of $S(A,B)$ we need to identify the following set:

A20 $$T\left(A,B\right):=\left\{\omega \ge M|{I}^{*}\left(A,\omega \right)={I}^{*}\left(B,\omega \right)\right\}.$$

This is the set of upper bounds for which $A$ has *the same* inequality as $B$. Because of the continuity of ${I}^{*}\left(.\right)$ with respect to $\omega$, the elements of $T$ separate the regions in the admissible space $\left[M,+\infty \right)$ for which $A$ exhibits higher inequality than $B$ and vice versa. Whenever the inequality comparison between $A$ and $B$ is completely robust, $T$ is the empty set $(\varnothing )$. Whenever ${I}^{*}\left(A,\omega \right)={G}^{*}\left(A,\omega \right)=G(A)/((\omega -{e}_{0}\left(A\right))/\omega )$, the condition imposed in (A7) can be written as

A21 $$G(A)/((\omega -{e}_{0}\left(A\right))/\omega ))=G(B)/((\omega -{e}_{0}\left(B\right))/\omega )).$$

Solving this equation in terms of $\omega$ gives a *unique* solution

A22 $${\omega }^{*}=\frac{{e}_{0}\left(B\right)G\left(A\right)-{e}_{0}\left(A\right)G\left(B\right)}{G\left(A\right)-G\left(B\right)} .$$

Since ${G}^{*}\left(A,\omega \right)$ is a monotonically decreasing function with respect to $\omega$, one must necessarily have that

A23 $$S\left(A,B\right)=\left\{\begin{array}{l}\left[M,+\infty \right) \mathrm{if}\, {\omega }^{*}<M \\ \left[M,{\omega }^{*}\right]\mathrm{ or }\,[{\omega }^{*},+\infty )\, \mathrm{if} \,{\omega }^{*}\ge M \end{array}\right..$$

## Appendix 4

Since ${G}^{*}\left(A,\omega \right)=G(A)/((\omega -{e}_{0}\left(A\right))/\omega ))$, one has that

A24 $$\underset{\omega \to \infty }{\mathrm{lim}}{G}^{*}\left(A,\omega \right)=\underset{\omega \to \infty }{\mathrm{lim}}\frac{\omega G(A)}{\omega -{e}_{0}\left(A\right)}=\frac{+\boldsymbol{\infty }}{+\boldsymbol{\infty }}.$$

To solve that indeterminacy, we apply L’Hôspital’s rule to the last expression, that is

A25 $$\underset{\omega \to \infty }{\mathrm{lim}}\frac{\omega G(A)}{\omega -{e}_{0}\left(A\right)}=\underset{\omega \to \infty }{\mathrm{lim}}\frac{\partial (\omega G(A))/\partial \omega }{\partial (\omega -{e}_{0}\left(A\right))/\partial \omega }=\frac{G(A)}{1}=G\left(A\right),$$

as we wanted to prove. Obviously, the same conclusion is reached when using the alternative definition of the normalized Gini index, i.e., ${\mathrm{AID}}^{*}\left(A,\omega \right).$ In that case, one has that

A26 $$\underset{\omega \to \infty }{\mathrm{lim}}{\mathrm{AID}}^{*}\left(A,\omega \right)=\underset{\omega \to \infty }{\mathrm{lim}}\frac{\omega \mathrm{AID}(A)}{{e}_{0}\left(A\right)(\omega -{e}_{0}\left(A\right))}=\frac{+\boldsymbol{\infty }}{+\boldsymbol{\infty }}.$$

To solve that indeterminacy, we apply L’Hôspital’s rule to the last expression, that is

A27 $$\underset{\omega \to \infty }{\mathrm{lim}}\frac{\omega \text{AID}(A)}{{e}_{0}\left(A\right)(\omega -{e}_{0}\left(A\right))}=\underset{\omega \to \infty }{\mathrm{lim}}\frac{\partial (\omega \text{AID}(A))/\partial \omega }{\partial ({e}_{0}\left(A\right)(\omega -{e}_{0}\left(A\right)))/\partial \omega }=\frac{\text{AID}(A)}{{e}_{0}\left(A\right)}=G\left(A\right),$$

as we wanted to demonstrate.

## Appendix 5

See Fig. 10, 11, 12 and 13.

## Footnotes

1 While there are important and well-documented exceptions to this association, they are usually circumscribed to specific population subgroups (like low SES groups; see Brønnum-Hansen, 2017, Permanyer et al., 2018; Sasson 2016; Van Raalte et al., 2014), or to very specific periods of time (e.g. the fall of Communism in Eastern Europe since 1989; see Aburto and Van Raalte 2018).

2 $A, B$ or $C$ can be age-at-death distributions of either sex, any country, and time.

3 Examples of absolute inequality measures include the variance, the standard deviation, the standard deviation around (or beyond) the modal age at death, the average inter-individual difference or the ${e}^{\dagger}$ coefficient. Relative inequality measures include the Gini coefficient, the Theil index, the mean log deviation, the coefficient of variation or the life table entropy $H$.

4 The compression-rectangularization hypothesis suggests that as the epidemiological transition unfolds, the human survival curves gradually become more rectangular with decreasing levels of mortality.

5 We have also investigated the consequences of choosing year-specific longevity records (i.e., allowed $\omega$ to change over time). The findings of the paper are largely unaffected.

6 Recall that normalizing the absolute and relative versions of the different inequality indices discussed here lead to the same inequality measure [see Eqs. (4, 5)].

7 The countries included in the database have remained highly stable since the 1990s.

8 Unlike the case of humans, maximal lifespans across the tree of life are not disputed, but they can vary tremendously [see the ‘Animal Aging and Longevity Database’ (AnAge) and Carey & Judge’ book (2002)]—an issue that could potentially compromise the comparability of the corresponding lifespan inequalities.

9 Admittedly, letting $\omega$ to attain the value of 1000 does not look very realistic. The main goal of our sensitivity analysis is to investigate the behavior of the normalized measures rather than making reasonable predictions about the future limits of human lifespan. Indeed, these is a heated, unsettled debate regarding the value of $\omega$, e.g., whether it exists, or how big it is (Dong et al., 2016; Lenart and Vaupel 2017). No matter what the conclusions of this crucial debate are, the ideas and results shown in this paper remain valid and applicable.

10 Several studies have identified increasing health inequalities across SES groups within countries, whereby socially disadvantaged groups tend to live shorter lives and experience higher uncertainty in the timing of death (Brønnum-Hansen 2017; Deboosere et al., 2009; Permanyer et al., 2018; Sasson 2016; Steingrímsdóttir et al., 2012; Tarkiainen et al., 2012; Van Raalte et al., 2014, 2018). In addition, countries with higher levels of economic inequality are lagging behind other counties with otherwise similar characteristics (as is the case with the US, UK, or Scotland; see Case and Deaton 2015; Leon et al., 2019), a worrisome pattern in a world with increasing within-country inequality.
